# Accelerated aortic plaque imaging using small field of view imaging and quadruple inversion recovery magnetization preparation

**DOI:** 10.1186/1532-429X-13-S1-P368

**Published:** 2011-02-02

**Authors:** Sarah A Peel, Tarique Hussain, Marina Cecelja, Abeera Abbas, Philip Chowienczyk, Matthew Waltham, Gerald Greil, René M Botnar

**Affiliations:** 1 King's College London, London, UK

## Introduction

Autopsy data has demonstrated that the abdominal aorta offers a valuable early window for the study of atherosclerosis. Current MRI strategies often suffer from long scan times or require slice gaps. We sought to implement a small field-of-view approach (zoom imaging) (Buecker, A. et al, 1998), to allow complete coverage of the thoracic and abdominal aorta. To improve blood signal nulling before and after contrast administration for identical imaging parameters, we combined zoom imaging with a quadruple inversion recovery (QIR) pre-pulse (Yarnykh, V. et al, 2002).

## Purpose

To demonstrate the feasibility of the novel zoom QIR technique for plaque imaging in the abdominal aorta.

## Methods

The QIR pre-pulse consists of a double inversion recovery (DIR) pre-pulse and a time delay (TI1) followed by a second DIR pre-pulse and a second time delay (TI2). Simulations in MATLAB were performed to study the steady-state longitudinal magnetization of blood (Mz_b_) for T1 values up to 2000ms. The effective TR was set to equal the RR-interval for heart-rates between 30 and 120bpm at 5bpm intervals. For each heart-rate, the optimal TI1 and TI2 values were calculated by minimizing the integral of Mz_b_ between 200 and 1400ms.

Six subjects were imaged on a 1.5T scanner using a 32-channel coil. Firstly pre-contrast images were acquired of the abdominal aorta using a 2D zoom DIR fast spin echo (TSE) sequence (33 slices, slice thickness=5mm, FOV=79x201mm, spatial resolution=1mmx1mm, TE=5ms, NSA=2, shortest trigger delay, imaging every other heartbeat and TI=400ms. Imaging time/slice≈11s)

Five selected slices were then imaged with the DIR pre-pulse replaced by the QIR pre-pulse with imaging parameters maintained (except imaging performed every heartbeat, five start-up cycles used to achieve a steady-state and imaging time/slice≈7s.) Approximately 10-15 minutes after the injection of a double dose of Gadovist, the zoom 2D DIR-TSE and QIR-TSE sequences were repeated in the same five slices.

## Results

**Figure 1:**
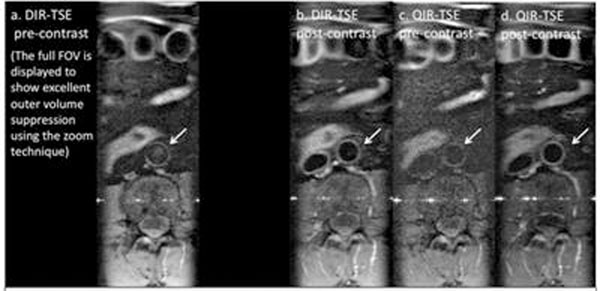
Images of the abdominal aortic wall (arrows) in a healthy volunteer: a. DIR-TSE (pre-contrast). B. DIR-TSE (post-contrast), c. QIR-TSE (pre-contrast) and d. QIR-TSE (post-contrast).

**Figure 2:**
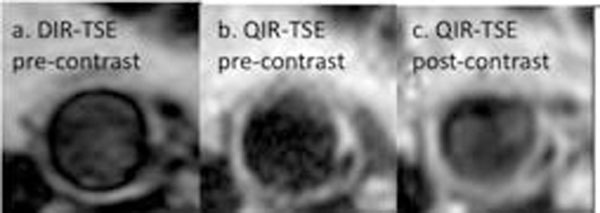
Images of volunteer with a plaque in the abdominal aorta. a. DIR-TSE (pre-contrast), b. QIR-TSE (pre-contrast), c. QIR-TSE (post-contrast).

Zoom imaging allowed a reduced field-of-view without wrap-around artifact and the QIR pre-pulse achieved blood signal nulling before and after contrast administration using identical imaging parameters in all volunteers (an example is shown in figure 1). One volunteer had an abdominal aortic plaque. The plaque is well defined in QIR-TSE images and there is evidence of post-contrast enhancement (see figure 2).

## Conclusions

The QIR pre-pulse has successfully been combined with ECG gating and zoom imaging. Preliminary data has shown that direct comparison of pre and post contrast imaging of abdominal aortic plaques is feasible.

